# Optimization of Instant Beverage Powder Containing Propolis Extract Nanoliposomes

**DOI:** 10.1155/ijfo/9099501

**Published:** 2024-12-09

**Authors:** Mehdi Mirzazadeh, Hadiseh Bagheri, Fatemeh Rasi, Nasim Mirzazadeh, Zahra Alam, Sahar Akhavan-Mahdavi

**Affiliations:** ^1^Department of Food Science and Technology, Faculty of Agriculture, Kermanshah Branch, Islamic Azad University, Kermanshah, Iran; ^2^Department of Food Science and Technology, Sari Branch, Islamic Azad University, Sari, Iran; ^3^Department of Food Science and Technology, Gorgan University of Agricultural Sciences and Natural Resources, Gorgan, Iran; ^4^Department of Food Science Engineering, Islamic Azad University Pharmaceutical Sciences Branch, Tehran, Iran; ^5^Department of Chemistry, Faculty of Science, Imam Khomeini International University, Qazvin, Iran

**Keywords:** drink, encapsulation, fortification, nanoliposome, propolis, RSM

## Abstract

Propolis is a natural resinous complex mixture produced by honeybees that contain various bioactive compounds. However, these bioactive compounds are chemically unstable and their absorption in the gastrointestinal tract is influenced by their solubility and stability. Encapsulation technology has been employed to increase their bioavailability and protect them against hostile conditions. Nanoliposomes are nanoscale lipid-based vesicles that can encapsulate various bioactive compounds, including propolis extracts. Therefore, in this study, propolis extract was encapsulated by nanoliposome technique and used in instant drink formulation. Nanoliposome characterization was done regarding particle size (255 ± 0.21 nm), zeta potential (−37.6 ± 1.14 mV), and encapsulation efficiency (73.71 ± 0.94). Response surface methodology (RSM) was employed to determine the effect of nanoliposome concentration (0%–5%) on the beverage characteristics including Brix, acidity, hygroscopicity, water solubility index, total phenol content, total microbial count, and sensory analyses. RSM predicted that a 3.19% nanoliposome would provide the overall optimum region for preparing the beverage with the best characteristics. Therefore, nanoliposome containing propolis can be successfully used in the enrichment of the beverage formulation by maintaining the sensory characteristics and improving its quality.

## 1. Introduction

Propolis is a resin compound collected by bees from the stems and leaves of plants and mixed with wax and enzymes of the bee's salivary glands. Propolis is a gummy, sticky substance, and its color varies from yellow–brown to dark brown depending on its source and how long it has been stored. It also has a sharp smell and strong taste. Propolis dissolves in varying amounts in organic solvents such as ethyl alcohol and acetone. Its chemical composition typically consists of 50%–55% gum and resin, 25%–30% wax, 10% essential oils or volatile fats, 5% pollen, and 5% organic compounds and mineral substances. [1]. The ratio and chemical compounds in propolis are directly related to the geographical location of the hive, the vegetation of the area, and the species of bee. So far, more than 300 active biological compounds have been isolated from propolis. Phenolic compounds, esters, flavonoids, terpenes, steroids, aromatic aldehydes, and alcohols are the most important compounds of propolis. Propolis is composed of a complex mixture of bioactive compounds that contribute to its therapeutic properties. Flavonoids, such as quercetin and kaempferol, possess antioxidant effects, protecting against oxidative stress. Phenolic acids, including caffeic acid and ferulic acid, contribute to antimicrobial activity. Terpenes present in propolis exhibit anti-inflammatory properties. Understanding these constituents is crucial for evaluating the potential of propolis in food fortification [2]. The use of propolis dates back to 3000 BC. The biological properties of propolis have been the focus of various researchers around the world for decades. Among the biological properties of propolis, we can mention antimicrobial, antifungal, antiparasitic, antiviral, anti-inflammatory, anticancer, and antioxidant properties [3]. Although propolis holds potential for use in food fortification, there are various challenges that must be confronted. The composition of propolis is subject to variability influenced by geographic and botanical factors, posing difficulties in standardization. Moreover, careful consideration is necessary to evaluate the potential influence of propolis on the sensory characteristics of fortified foods to ensure consumer acceptance. On the other hand, one of the main problems of using raw propolis or ethanol extract is its lack of stability in the water environment. This problem has challenged the biomedical applications of propolis. One of the ways to benefit more from bioactive substances in propolis and increase its stability in the aquatic environment is to encapsulate it or prepare structures with nanodimensions from it [2].

The process of encapsulation involves enclosing an active ingredient or a combination of substances, which may be small particles, liquid, or gas, within a protective coating or shell for subsequent release. Beyond safeguarding against external factors like oxygen, light, and heat, encapsulating active bioactive compounds offers additional benefits. It can enhance shelf life, prevent interference with product performance, and mask undesirable odors and flavors [4]. Due to its capacity to convert unstable products, encapsulation technology proves highly advantageous for the food industry. It serves as a vital method to meet all claims in the food industry, delivering bioactive food components precisely when and where needed. However, a significant challenge in employing fortified food products is ensuring the preservation of product integrity and maintaining the active ingredients until the point of consumption [5].

Shakoury et al. [6] encapsulated propolis extract at concentrations of 1%, 2.5%, and 4% by employing whey protein isolate (WPI) as the encapsulating material. This was prepared at pH levels of 3.2 and 7.5. The study revealed that higher propolis concentrations resulted in a more controlled release during gastrointestinal digestion. The microparticles exhibited favorable characteristics, indicating their potential as food additives in industrial food product applications [6].

A liposome is a spherical vesicle with at least one lipid bilayer that surrounds an aqueous (water) core. The structure of a liposome mimics the cell membrane, as it consists of a lipid bilayer with hydrophilic (water-attracting) heads facing outward and hydrophobic (water-repelling) tails facing inward. This arrangement allows liposomes to encapsulate drugs, nutrients, or other substances within their aqueous core or lipid bilayers [7, 8]. Nanoliposomes have gained interest in the food industry due to their unique properties, including their ability to encapsulate and deliver bioactive compounds. Some applications of nanoliposomes in the food industry include encapsulation of nutrients, flavor and aroma enhancement, functional ingredient delivery, fat replacement, improved stability of emulsions, and controlled release of additives [9]. Liposomes stand out as the most widely recognized vehicle for delivering propolis, having obtained generally recognized as safe (GRAS) status. These nanoparticles (NPs) are considered safe, biocompatible, and environmentally friendly; possess stability during storage; and exhibit controlled drug delivery systems, aiming at attaining the desired therapeutic response [2].

Ramli et al. [10] stated that liposomes can be effectively employed to safeguard the bioactive compounds in propolis, offering an efficient nanocarrier system capable of protecting these substances from unfavorable gastrointestinal conditions [10]. Aytekin et al. [11] explored the encapsulation of bioactive compounds from propolis using liposomes as a carrier. The formulated propolis–loaded liposomal system exhibited encouraging outcomes as a topical remedy for wounds, incorporating both antioxidant and antimicrobial effects [11].

Up to now, various bioactive compounds of nanoliposomes have been used in the fortification of different food products such as yogurt fortified with fish oil nanoliposomes [8], nanoliposome containing D_3_ in beverage fortification [12], fortification of skim milk with nanoliposomes loaded with shrimp oil [13], fortification of Indian curd with chia oil nanoliposome [14], and herbal extract–encapsulated nanoliposomes [15]. However, so far, no study has been done on the nanoencapsulation of propolis extract in food enrichment; therefore, the aim of this study is to produce nanoliposome containing propolis and investigate the nanoliposome characteristics as well as select the optimal concentration of nanoliposome for use in instant drink formulation.

## 2. Materials and Methods

### 2.1. Materials

Pure propolis was provided by a beekeeping farm (Gorgan, Iran). Ingredients of beverages were purchased from local markets. All other chemicals used in this study were of analytical grade and purchased from chemical suppliers.

### 2.2. Propolis Extraction

Extraction was performed according to Mirbagheri et al. [3]. To prepare a solution with a concentration of 10% weight/volume, 5 g of raw propolis was dissolved in 50 mL of 70% ethanol. The extraction process took place on a magnetic stirrer, running continuously for 24 h. Following filtration with Whatman No. 1 filter papers, the extracted propolis solution underwent concentration using a rotary evaporator. The resulting residue was subjected to lyophilization, and the dried crude extracts were stored in a sealed, opaque bottle at 4°C [3].

### 2.3. Preparation of Nanoliposomes

Nanoliposomes were prepared according to the method of Rasti, Erfanian, and Selamat [16] with some modifications. The liposomal formulation (lecithin with a concentration of 3% and oil) was mixed in a hot water bath at 30°C to dissolve the lecithin in the oil. At the end, this solution was hydrated by adding deionized water and 2% glycerol containing the desired extract with a concentration of 1% at 55°C and mixed with an Ultra-Turrax T-25 Digital Homogenizer (IKA, Germany) for 10 min. The liposomal dispersion was exposed to ultrasound (7 min: 1 min on and 1 min off) at a temperature of 25°C. Nanoliposomes were placed at 25°C (ambient temperature) for 1 h to become stable [16].

### 2.4. Characterization of Nanoliposomes Containing Propolis Extract

#### 2.4.1. Measurement of Particle Size, Polydispersity Index (PDI), and Zeta Potential

The average particle diameter, PDI, and zeta potential of nanoliposomes containing the extract were determined by the dynamic light scattering method by a NanoSizer 3000 laser refraction device (Malvern Instrument, England) at 25°C. For this purpose, samples were first diluted 50 times using distilled water. Then, the samples were transferred into the capillary tube by a syringe and the capillary tube was placed in the special place of the device [17].

#### 2.4.2. Encapsulation Efficiency (%EE)

One milliliter of the nanoliposome solution was transferred into an Amicon filter (molecular weight 100 kDa, Millipore, England) and centrifuged at a force of 2000 × *g* (relative centrifugal force (RCF)) for 10 min. The amount of uncoated compounds was collected at the end of Amicon filter. The absorbance of this part was read with an ultraviolet-visible spectrophotometer at a wavelength of 278 nm, and the concentration of the uncoated extract was determined using a spectrophotometer (PG Instruments Ltd, United States). %EE was calculated according to Equation (1) using the results from total phenol content (TPC) and surface anthocyanin content (SAC) [18].

(1) $$\begin{array}{c} \%\text{EE}=\frac{\left(\text{TPC}-\text{SAC}\right)}{\text{TPC}}\times 100 \end{array}$$

#### 2.4.3. Liposome Stability During 60 Days of Storage

The physical stability of the nanoliposomes was determined visually at ambient temperature (25°C), and their biphasing (precipitation formation) during 60 days of storage was done to check the stability of the liposomal system. Also, the chemical stability of the solution was calculated by measuring the amount of free and encapsulated propolis at ambient temperature (25°C) after 60 days according to the method in Section 2.4.2 [19].

#### 2.4.4. Morphology of Nanoliposomes With Transmission Electron Microscopy (TEM)

In order to investigate the morphology and microstructure of nanoliposomes, TEM and negative staining method were used. Micrographs using Philips CM20 TEM operating at 200 kV were generated and recorded by an Olympus TEM charge-coupled device (CCD) camera.

### 2.5. Preparation of Instant Beverage Powder

Milk powder–based flavored instant beverage powder is a product that is obtained from a mixture of instant milk powder, sugar, or other permitted edible sweeteners and permitted edible additives in the form of powder or grains. After dissolving in water, this product is solidified as a drink. The appearance of this product should be in the form of powder or grains and uniform and nonsticky and easily dissolve completely in water.

The drink ingredients contain instant milk powder (10 g), sugar (25 g), citric acid (0.5 mL), cream power (1 g), tricalcium phosphate (0.1 mL), guar gum (0.5 g), banana flavor (2.5 g), colorants (2 mL), and benzoate sodium solution 0.05% (0.1 mL). Nanoliposomes containing propolis extract was added according to the response surface methodology (RSM) (0%–5%). All the dry ingredients were combined, and then, the pH of the samples was adjusted to 4 using citric acid, and 0.05% sodium benzoate solution was added to them. Next, some boiling water was added to the drinks in order to completely dissolve the ingredients and reach the final volume of 100 mL. The prepared drink solutions were stored at 4°C.

### 2.6. Evaluation of Beverage Characteristics

#### 2.6.1. Physicochemical Characteristics

Physicochemical characteristics of beverage such as protein content [20], acidity [21], Brix (refractometer, Huixia, China), and color analysis (Lovibond colorimeter, CAM100, England) were evaluated after preparing drink samples. Then, the produced beverages were powdered using a freeze dryer (Operon, South Korea) at < −40°C for 20 h and hygroscopicity and the water solubility index (WSI) of the powders were measured using the method described by Akhavan Mahdavi et al. [22].

#### 2.6.2. Determination of TPC

The TPC of the beverage was assessed using the Folin–Ciocalteu micromethod. In a 50-mL volumetric flask, a mixture of 1 mL of a standard gallic acid solution, 6 mL of methanol, 2.5 mL of Folin–Ciocâlteu reagent, and 5 mL of 7.5% Na_2_CO_3_ was prepared. The final volume was adjusted with purified water. After overnight storage, spectrophotometric analysis was conducted at a wavelength of *λ* = 765 nm (PG Instruments Ltd, United States). A calibration curve using gallic acid (0–700 mg/mL) was established, and the TPC of both fig samples and canola oil was expressed in terms of gallic acid from this curve [23, 24].

### 2.7. Total Count of Microorganisms

To enumerate the total microorganism count, 1 mL of the diluted samples was transferred onto sterile plates. Subsequently, 15 mL of molten count agar medium was poured over the samples in the plates, and the plates were swirled in a rotary motion to ensure uniform distribution. After solidification of the culture medium, the plates were incubated at 30°C for 72 h. The count of cream-colored colonies was then determined [25].

### 2.8. Sensory Analyses

Twenty panelists with training, aged between 20 and 40 years, were chosen to participate in the sensory evaluation. The panel assessed crucial beverage attributes, including color, flavor, and overall acceptance, using a 5-point hedonic scale [23].

### 2.9. Experimental Design for RSM

RSM was utilized to explore the influence of various concentrations of nanoliposomes containing propolis (ranging from 0% to 5%) on beverage quality. The composition of variables was determined through optimal design, generating 13 experimental settings with one factor based on the principles of RSM (I-optimal) using Design-Expert 11.1.0.1 (StatEase Inc., Minneapolis, Minnesota) (refer to Table 1). The same software was employed for numerical and graphical optimizations. The characteristic polynomial equation for the I-optimal design based on the nanoliposome concentration factor is as follows:

 $$\begin{array}{c} y=\beta_{0+}\beta_{1}A+\beta_{2}A^{2}+\beta_{3}A^{3}+\beta_{4}A^{4}+\beta_{5}A^{5}+\epsilon \end{array}$$

where *y* represents the response (e.g., Brix and acidity), *A* represents the nanoliposome concentration, *β* values are the coefficients obtained through regression, and *ϵ* denotes the error term.

Randomization of experiments was carried out to minimize the impact of unexplained variability in observed responses due to external factors. The center point in the design was replicated six times to assess the repeatability of the method.

The desirability function was used to identify optimal conditions by converting multiple response variables into a single composite desirability score. This score ranges from 0 (least desirable) to 1 (most desirable). The generalized form of the desirability function DDD for individual responses is given by Equation (2):

(2) $$\begin{array}{c} D={\left(\overset{n}{\underset{i=1}{∐}}d_{i}^{\omega_{i}}\right)}^{\left(1/\sum \omega i\right)} \end{array}$$

where *d*_*i*_ represents the desirability of each response *i* and *ω*_*i*_ is the weight assigned to each response based on its importance. The goal was to maximize *D*, achieving the most balanced and desirable outcome for all measured attributes.

## 3. Results and Discussion

### 3.1. Nanoliposome Characterization

The particle size and PDI of nanoliposomes containing propolis extract are critical parameters that influence the stability, bioavailability, and release properties of encapsulated bioactive compounds. A smaller particle size enhances cellular uptake and improves stability by reducing gravitational settling and aggregation. In this study, the nanoliposomes exhibited an average particle size of 255 ± 0.21 nm, which is within the desirable range for food and pharmaceutical applications, as it supports both effective delivery and stability of bioactive compounds [26].

**Figure 1:**
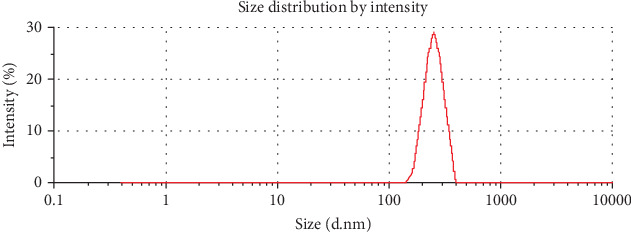
Particle size distribution of nanoliposome containing propolis extract.

The PDI value of 0.217 ± 0.04 reflects a narrow size distribution and indicates the homogeneity of the nanoliposome formulation, which is essential for consistent product quality. A low PDI (generally < 0.3) is preferred because it suggests uniform particle size, reducing the likelihood of instability due to polydispersity [27]. The results for particle size and PDI suggest that the formulated nanoliposomes are well suited for maintaining the integrity and controlled release of encapsulated propolis within the beverage matrix (Figure 1).

In general, several factors influence the size and dispersion of particles in colloidal liposome systems, including the structure of the active compound; the type and concentration of compounds used in the formulation; the arrangement and structure of the membrane; the type and concentration of stabilizers and microencapsulated active substances; the lipid bilayer composition; the ratio of phospholipids to the active substance; the preparation and production method of the liposomes; and process conditions such as stirring speed, type of membrane stabilizer, duration, and temperature. The differences observed in the size and dispersion of particles between the results of our research and the research of other researchers can be related to these mentioned cases [28, 29].

**Figure 2:**
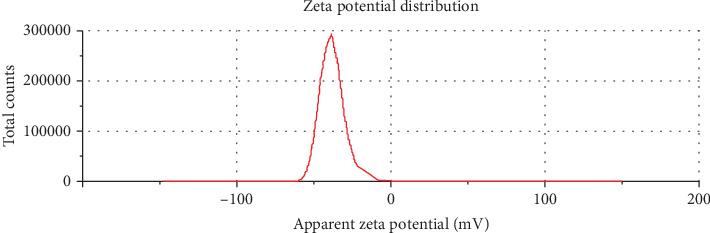
Zeta potential of nanoliposome containing propolis extract.

In this research, the results of electrophoretic mobility and zeta potential were used in order to evaluate the action of electrostatic repulsion forces between charged particles such as nanoliposomes and to evaluate the stability of vesicular suspensions as well as the binding of liposomes to the membrane of target cells. In fact, the zeta potential is the total charge of a particle in the liquid environment or the potential difference between the mobile ion layer and the nonmobile layer, and it is considered one of the most important factors for determining the electrical state of the surface of colloidal solutions. The measurement of this factor is useful in controlling the aggregation and precipitation of nanoliposomes, which are important factors in the stability of nanoliposomes [30, 31]. In addition, it is an important parameter in the binding of liposomes to the cell membrane and the binding of active substances to the liposome and their release rate. The higher the value of this factor (whether positive or negative), the greater the repulsion between the particles, and as a result, it reduces the adhesion of the particles to each other and the stability of the colloidal systems is provided. The nanoliposome produced had a negative zeta potential and a surface charge of −37.6 ± 1.14 mV, which indicates the high electrostatic repulsion force of nanoliposomes is their stability and prevention of aggregation, coagulation, and flocculation over time (Figure 2).

%EE is one of the important indicators that shows the efficiency of NPs to maintain bioactive compounds in their structure. Increasing efficiency is important from the aspect of reducing costs and improving effectiveness. The %EE of the extract rich in propolis extract in nanoliposome was 73.71 ± 0.94. However, some researchers reported different results in this regard. The efficiency in this study was higher than the efficiency reported by Pinilla and Brandelli [32] and Erami, Amiri, and Jafari [33] for nanoliposomes containing garlic extract (47%) and bitter gourd (*Momordica charantia*) fruit extract (70), respectively [32, 33]. Mohammadi, Ghanbarzadeh, and Hamishehkar [12] reported the efficiency of vitamin D encapsulation with nanoliposomes in the range of 93% for all formulations [12]. Wu et al. [34] reported 75.36% efficiency and 245.6-nm particle size in the production of nanoliposomes containing lysozyme under optimal conditions [34]. The reason for this difference in efficiency in different studies can be seen in the factors affecting the efficiency of encapsulation. The efficiency of microcoating is influenced by several factors, including the nature of the active substance (its lipophilicity or hydrophilicity and solubility); the characteristics of the phospholipid (such as type, fatty acid length and arrangement, and saturation); the ratio of phospholipid to the microcoated compound; the preparation and production method of nanoliposomes; the concentration and type of stabilizers, such as cholesterol; and environmental conditions, including temperature, pH, and ionic strength [35–37].

**Figure 3:**
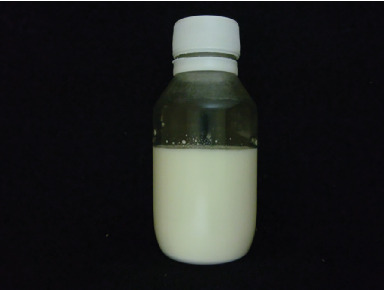
Physical stability of nanoliposome solution containing propolis during 60 days of storage at ambient temperature (25°C).

The physical stability of the nanoliposome solution is presented in Figure 3. According to Figure 3, it is clear that the propolis nanoliposomal solution showed good stability during storage at room temperature (25°C). The smaller size of the particles, the stiffness of the membrane, and the improvement of the zeta potential due to the use of optimal amounts of cholesterol as well as the creation of high electrostatic repulsion can be considered the main reasons for this. In this investigated sample, biphasing and formation of sediment were observed to a very minor extent on the 60th day of storage (Figure 3). Due to the small density difference between the phospholipids (liposomal bilayers) and the continuous phase (aqueous medium), gravitational separation in liposomes is very slow and does not occur often. One of the main reasons for the physical instability of liposomal systems is the merging of liposomal bilayers due to their collision with each other and the fusion of liposomal membranes. The physical stability of liposomes depends on several factors, such as the average particle size, the number of layers, the phospholipid structure, and the method used to produce the liposomes [38, 39].

**Figure 4:**
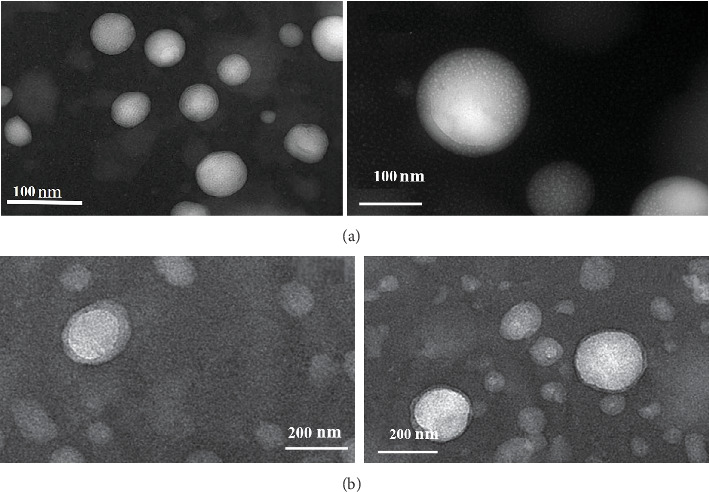
TEM images of nanoliposomes (a) without propolis extract and (b) loaded with propolis extract.

TEM images provide visual information about the size morphology and particle size distribution of nanoliposomes. TEM images (Figure 4) show that the unloaded nanoliposome (Figure 4(a)) and the content of propolis extract (Figure 4(b)) had a spherical and quasispherical appearance and their particle size is in the nanorange. Also, the images show that the presence of loaded propolis extract had a favorable effect on the compression of phospholipid bilayers and improved the spherical shape of the vesicles. The bilayer nature of nanoliposome is well evident in this figure. Rapeseed lecithin used in this study was not a pure phospholipid, and a series of lipid compounds formed the mentioned lecithin structure. The small presence of oil droplets in the colloidal suspension of nanoliposomes is well shown in TEM images (bright spots in Figure 4(b)).

### 3.2. Beverage Characterization Optimization

The outcomes from the specified series of experiments are outlined in Table 2. According to the analysis of variance (ANOVA), the concentration of nanoliposomes (*A*) was identified as a significant factor affecting the Brix of the beverage. The coefficients of the model derived from regression analysis of the experimental data are presented in Equation (3):

(3) $$\begin{array}{c} \text{Brix}=10.2365+1.60069\;A+2.81433\;A^{2}+0.49735\;A^{3}+-1.96565\;A^{4} \end{array}$$

Brix or total dissolved solids in the drink were obtained in the range of 9 to 13.5, which is consistent with the standard amount in instant drink powders. By increasing the concentration of added nanoliposome, the Brix content of the drink increased.

This adjustment yielded a more accurate fit for the data, as confirmed by a significant reduction in the lack-of-fit value. The revised model now provides better predictive accuracy for Brix in relation to the concentration of nanoliposomes, enhancing the reliability of our findings.

According to the ANOVA, the concentration of nanoliposomes (*A*) was identified as a significant factor in impacting the acidity of the beverage. The coefficients of the model, derived through regression analysis of the experimental data, are presented in the following equation:

(4) $$\begin{array}{c} \text{Acidity}=3.28971+0.416386\;A+1.30034\;A^{2}+-1.83929\;A^{3}+-4.51269\;A^{4}+1.3707\;A^{5}+3.17493\;A^{6} \end{array}$$

Based on ANOVA, nanoliposome concentration (*A*) was a significant factor in influencing the hygroscopicity of the beverage. The corresponding coefficients of the model obtained by regression analysis of the experimental data are shown in the following equation:

(5) $$\begin{array}{c} \text{Hygroscopicity}=61.7295+-5.77109\;A+-4.73339\;A^{2} \end{array}$$

Hygroscopicity refers to the ability of a substance to absorb and retain moisture from the surrounding environment. In the context of instant powders, which are often used in food and beverage products, the hygroscopic nature of the powder can have several important implications such as clumping and caking prevention, storage stability, flowability and handling, dissolvability and reconstitution, and quality control. Hence, understanding and managing the hygroscopic nature of instant powders are crucial for ensuring product quality, shelf life, and consumer satisfaction. Manufacturers must strike a balance to minimize moisture-related issues while maintaining the desired characteristics of the final product.

**Figure 5:**
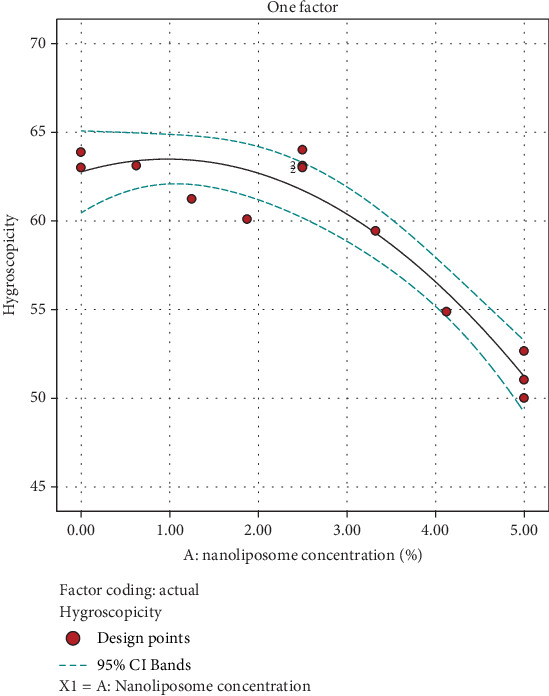
Effect of nanoliposome concentration added to hygroscopicity of instant powder.

According to Figure 5, the powder samples without nanoliposome have higher hygroscopicity than the samples containing nanoliposome. On the other hand, with the increase in the concentration of added nanoliposome, the hygroscopicity amount decreases, which shows the positive effect of encapsulation in protecting propolis and reducing moisture penetration.

According to the ANOVA, both the concentrations of nanoliposomes (*A*) were recognized as significant factors affecting the WSI of the beverage. The coefficients of the model, obtained through regression analysis of the experimental data, are presented in the following equation:

(6) $$\begin{array}{c} \text{WSI}=5.29203+-0.28109\;A+3.43741\;A^{2}+-0.0899537\;A^{3}+-1.97653\;A^{4} \end{array}$$

**Figure 6:**
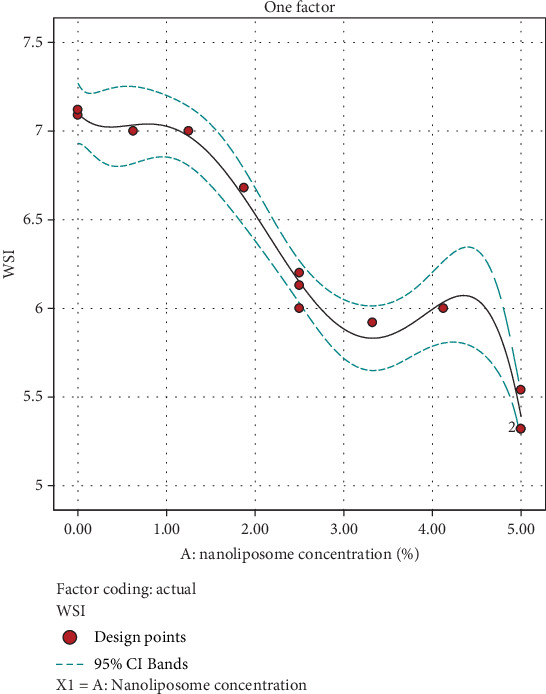
Effect of nanoliposome concentration added to the water solubility index (WSI) of instant powder.

The WSI of instant powder is a critical parameter that indicates the ability of a powder to dissolve in water. A high WSI ensures that the powder dissolves rapidly in water, allowing for convenient and efficient reconstitution. This is particularly important for consumer products where convenience and speed of preparation are key factors [40]. A consistent and high solubility index ensures that consumers get the same quality and taste with each use, reducing the likelihood of clumps or uneven distribution of ingredients. Moreover, powders with good water solubility are less likely to clump or form lumps during storage, contributing to better shelf stability and maintaining product quality throughout its shelf life [39, 41]. As shown in Figure 6, the control treatment without nanoliposome had the highest WSI. Also, with the increase in the concentration of nanoliposome, this index decreased, which can be attributed to the hydrophobic nature of lipids that make up the wall and the low solubility of propolis in water. Akhavan Mahdavi et al. [22] reported high amounts of WSI of the powder in the production of jelly powder containing encapsulated anthocyanin, which was due to the hydrophilic nature of anthocyanin [22], while Shaddel and Rajabi-Moghaddam [39] reported lower values of this index due to the hydrophobic nature of caffeine in the production of drink formulation containing caffeine in chitosan-coated nanoliposomes.

According to ANOVA, the concentration of nanoliposomes (*A*) was identified as a significant factor affecting the total phenols of the beverage. The coefficients of the model, obtained through regression analysis of the experimental data, are presented in the following equation:

(7) $$\begin{array}{c} \text{TPC}=99.817+42.5471\;A+-14.1497\;A^{2}+-51.8391\;A^{3}+42.0177\;A^{4}+75.2476\;A^{5} \end{array}$$

The TPC of propolis can vary widely depending on factors such as the geographical location where the propolis is harvested, the plant sources available to the bees, and the specific extraction methods used. The TPC is often measured as gallic acid equivalents (GAE) per gram or milligram of propolis. Different studies report varying values for the TPC of propolis [42, 43]. The majority of the antioxidant properties of propolis were assigned to galangin and pinocembrin. It was acknowledged that phenolic compounds in propolis contribute hydrogen ions to free radicals, thereby impeding the oxidation of lipids, proteins, and nucleic acids [44].

**Figure 7:**
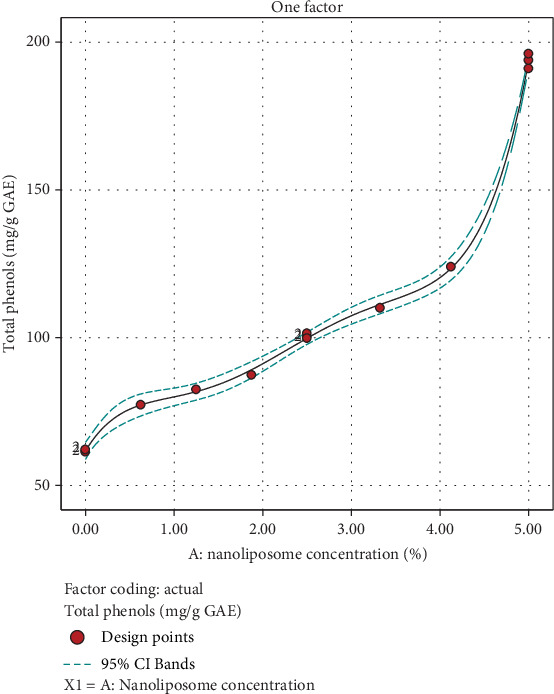
Effect of nanoliposome concentration added to total phenol content of instant powder.

The results showed that the concentration of nanoliposome added to the drink has a direct effect on the increase of total phenolic compounds, so that the treatments without nanoliposome had the lowest amount and the treatments with high concentration of nanoliposome had more phenol (Figure 7).

According to ANOVA, the concentration of nanoliposomes (*A*) was identified as a significant factor impacting the microbial total count of the beverage. The coefficients of the model, derived through regression analysis of the experimental data, are presented in the following equation:

(8) $$\begin{array}{c} \text{Microbial total count}=2313.28+-833.326\;A+654.752\;A^{2}+25.6163\;A^{3}+-787.046\;A^{4} \end{array}$$

**Figure 8:**
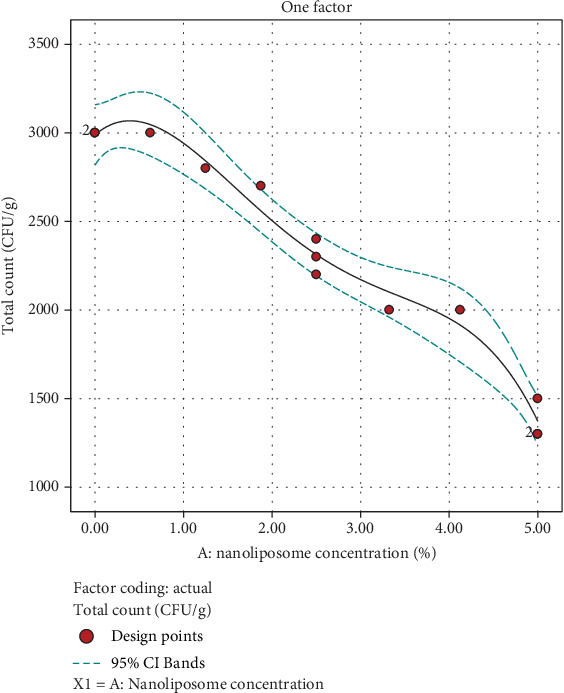
Effect of nanoliposome concentration added to total microbial count of instant powder.

Propolis is well known for its antibacterial activity, and this property is attributed to the presence of various bioactive compounds, including polyphenols, flavonoids, terpenes, and other substances. The antimicrobial effects of propolis have been demonstrated against a broad spectrum of bacteria. Propolis has been used traditionally for wound healing, and its antibacterial properties play a role in preventing and treating infections. It can be applied topically to wounds to reduce the risk of bacterial contamination [45, 46]. The results showed that by increasing the concentration of nanoliposome, the total bacterial count of drinks decreased after storage, demonstrating a 4.57-log reduction. (Figure 8).

In our study, as nanoliposome concentration increased, the microbial total count in the beverage decreased significantly, demonstrating a log reduction of approximately 4.5 log. Although this reduction falls slightly below the 5-log threshold typically sought in food safety applications, it effectively enhances microbial stability and product safety over storage time. Further optimization could involve increasing acidity or incorporating additional preservation strategies to achieve or exceed the 5-log microbial reduction threshold, which is desirable for industrial applications. The results indicate that propolis-loaded nanoliposomes are a promising means of fortifying beverages with both antimicrobial and health-promoting bioactive compounds.

The total microbial count obtained in this study complies with the microbial limits for powdered beverages set by international food safety guidelines, such as the European Union Regulation (EC) No. 2073/2005 on microbiological criteria for foodstuffs [47], and national standards including Institute of Standards and Industrial Research of Iran (ISIRI) No. 2395 for instant drink powders [48]. Specifically, the optimized formulation with 3.19% nanoliposome concentration consistently yielded microbial counts well within the acceptable limits for safe consumption. Further, parameters such as acidity, which can influence microbial stability, align with beverage quality guidelines from bodies like the Codex Alimentarius and FDA, which advocate for a pH below 4.6 to inhibit pathogenic growth. Our optimized beverage formulation achieved an acidity level compliant with these guidelines, enhancing product safety [49, 50].

According to ANOVA, both the concentrations of nanoliposomes (*A*) were recognized as significant factors affecting the sensory analyses of the beverage. The coefficients of the model, obtained through regression analysis of the experimental data, are presented in the following equation:

(9) $$\begin{array}{c} \text{Flavor}=4.08666+-0.471965A \end{array}$$

(10) $$\begin{array}{c} \text{Color}=4.26741+0.473797\;A+1.3874\;A^{2}+-0.512391\;A^{3}+-1.14992\;A^{4} \end{array}$$

(11) $$\begin{array}{c} \text{Overall acceptance}=4.03169+-0.327168\;A+-0.258328\;A^{2} \end{array}$$

Food manufacturers need to conduct sensory evaluations during the development of propolis-enriched products. This involves assessing the taste, aroma, appearance, and overall sensory experience of the food to ensure that the addition of propolis aligns with the intended characteristics of the product and meets consumer expectations. The sensory effects of adding propolis to food formulations can vary depending on factors such as the concentration of propolis, the type of food product, and the specific characteristics of the propolis used. Propolis has a distinctive taste that can be described as bitter, astringent, or resinous. The addition of propolis to food formulations may impart these flavor characteristics to the final product. Despite the addition of banana-flavored essential oil to the drink formulation, the distinct aroma and taste of propolis had an adverse effect on the taste of the samples so that the panelists assigned lower points to drinks containing high concentrations of propolis. Propolis can impart a brownish color to food products. However, the freeze-dried propolis extract had a creamy color. Therefore, it did not have much effect on the color of the final drink. Generally, the panelists gave a similar score between samples without nanoliposomes and samples containing lower concentrations of nanoliposomes containing propolis, which indicates the protective and covering effect of nanoliposomes in masking distinctive flavor and color of the propolis.

### 3.3. Optimization

In this study, the optimization process assigned specific goals and importance levels to each response variable based on their impact on the final product. For instance, hygroscopicity was minimized to enhance shelf stability by reducing moisture absorption, while total phenols were maximized to improve the antioxidant properties. Flavor and color were prioritized (importance level = 5) to ensure high consumer acceptance and visual appeal, which are essential attributes for a market-ready beverage. By defining these goals and weights, the RSM was tailored to balance functional and sensory qualities, thereby aligning the final product with industry standards and consumer preferences.

**Figure 9:**
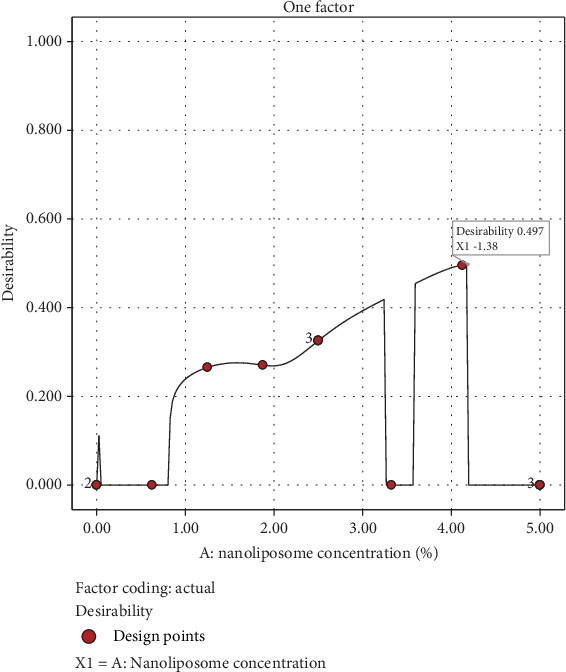
Desirability plot illustrating the optimal nanoliposome concentration for maximizing beverage quality characteristics.

The RSM was applied to evaluate the effect of nanoliposome concentration on various beverage attributes. Using desirability functions as the optimization criterion, the ideal concentration of nanoliposome was found to be 3.19%, with a corresponding desirability value of 0.139 (Figure 9). This concentration achieves an optimal balance across the targeted beverage characteristics as listed in Table 3. For validation, a confirmation experiment was conducted at this optimized condition, and the results were consistent with the model predictions, yielding a high *R*^2^ value of 0.99. This confirmed the model's accuracy and reliability. Additionally, a generalized form of the desirability function equation can be provided in the Materials and Methods section to further explain the optimization approach.

## 4. Conclusions

In recent years, there has been a growing consumer interest in understanding the influence of food on health. Considering the established link between bioactive compounds, health, and disease and acknowledging the widely recognized health-promoting effects of propolis and its encapsulated forms in terms of protecting, increasing stability, reducing unpleasant aroma and taste, and targeted release of bioactive compounds, in this research, the characteristics of nanoliposomes containing propolis and its optimization in addition to beverage formulations were investigated. The optimization results showed that the overall optimum region with the best characteristics was found to be at 3.19%. The results indicated the success of enriching the instant drink powder formulation with the optimal amount of nanoliposomes and, as a result, the production of functional food by improving its physicochemical, microbial, and sensory characteristics.

## Figures and Tables

**Table 1:** Optimal experimental design matrix.

**Run**	**Nanoliposome concentration (%)**
1	1.25
2	5.00
3	5.00
4	0.00
5	4.13
6	2.50
7	1.88
8	5.00
9	0.00
10	0.63
11	2.50 (center point)
12	3.33
13	2.50 (center point)
14	2.50 (center point)
15	1.25
16	2.50 (center point)
17	5.00
18	2.50 (center point)
19	2.50 (center point)
20	1.25

**Table 2:** Variance analysis of the second-order polynomial regression model for determination of scaffold characteristics.

**Source**	**p** ** value**								
	**Brix**	**Acidity**	**Hygroscopicity**	**WSI**	**Total phenols**	**Microbial total count**	**Flavor**	**Color**	**Overall acceptance**
Model	< 0.0001	0.0278	< 0.0001	0.0003	< 0.0001⁣^∗^	< 0.0001	< 0.0001	0.0003	0.0002
*A*—nanoliposome concentration	0.0025	0.0216	< 0.0001	0.4714	0.0176	0.0005	< 0.0001	0.0007	0.0002
*A* ^2^	0.0114	0.0226	0.0013	0.0038	< 0.0001⁣^∗^	0.0915		0.0001	0.0224
*A* ^3^	0.1910	0.0119		0.8320	0.0755	0.8799		0.0008	
*A* ^4^	0.0367	0.0131		0.0392	0.0014⁣^∗^	0.0399		0.0003	
Lack of fit	0.2150	0.4555	0.0503	0.0028	0.9053⁣^∗^	0.3209	0.4282	0.3858	0.2267

⁣^∗^A statistically significant difference with a *p* value < 0.01, indicating strong evidence against the null hypothesis at a 1% significance level.

**Table 3:** Optimization criteria used in this study.

**Variable**	**Goal**	**Importance**
Brix	In range	3
Acidity	In range	3
Hygroscopicity	Minimize	5
WSI	Maximize	5
Total phenols	Maximize	5
Microbial total count	Minimize	5
Flavor	Maximize	5
Color	Maximize	5
Overall acceptance	Maximize	5

## Data Availability

Data will be made available upon request to the corresponding author.
